# Detection of previously undiagnosed conditions in midlife preventive health examinations

**DOI:** 10.1038/s41598-026-53658-2

**Published:** 2026-06-03

**Authors:** Linda Kalski, Lorena Hafermann, Tilman J. Pulst Caliman, Franziska Greiß, Athanasios Karathanos, Charleen Pächter, Carolin Herrmann, Bernd Wolfarth

**Affiliations:** 1 Institute of Sport Science, Humboldt-Universität Zu Berlin, Berlin, Germany; 2 Department of Sports Medicine, Charité – Universitätsmedizin Berlin, Berlin, Germany; 3 Institute of Biometry and Clinical Epidemiology, Charité – Universitätsmedizin Berlin, corporate member of Freie Universität Berlin and Humboldt-Universität Zu Berlin, Berlin, Germany; 4 Mathematical Institute, Heinrich Heine University Düsseldorf, Düsseldorf, Germany

**Keywords:** Screening, Checkup, Diagnoses, Prevention, Examination, Cardiology, Diseases, Endocrinology, Health care, Medical research, Risk factors

## Abstract

This study aimed to investigate the prevalence of previously undocumented suspected diagnoses identified during preventive health examinations in middle-aged adults, and to examine their associations with clinical and behavioral factors. In this cross-sectional study, 1,040 adults aged 45–59 years underwent preventive clinical examinations comprising self-reported medical histories, anamneses, previous medical records, and laboratory tests (LDL cholesterol, blood pressure, HbA1c, TSH). Suspected diagnoses were defined by clinical thresholds (e.g., LDL > 116 mg/dl, hypertension ≥ 140/90 mmHg, HbA1c > 6.0%). Multivariable logistic regression analyzed associations between multiple variables and the three binary outcomes, any suspected diagnosis of hypertension and elevated cholesterol. The mean age of participants was 52.93 years (sd = 4.17), with 61% male (n = 631). In total, 421 (41%) had at least one newly identified suspected diagnosis. The most frequent findings were elevated LDL cholesterol (n = 287, 28%) and hypertension (n = 93, 9%). Male sex was associated with higher odds of any suspected diagnosis (OR: 1.42; 95%-CI: 1.09–1.85) and having hypertension (OR: 2.24; 95%-CI: 1.31–3.99). BMI was associated with hypertension (OR: 1.15; 95%-CI: 1.08–1.21) and showed a weak association with elevated cholesterol (OR: 1.04; 95%-CI: 1.00–1.08). A recent blood test was linked to lower odds of any suspected findings (OR: 0.65; 95%-CI: 0.50–0.84). No consistent associations were observed for lifestyle factors. A family history of dyslipidemia was associated with elevated cholesterol (OR: 1.55; 95%-CI: 1.01–2.40). Preventive health screenings can reveal a substantial number of previously undiagnosed conditions, particularly among individuals with limited healthcare contact or elevated risk. The findings suggest that such screenings may offer a significant value for targeted risk groups in midlife. Further research should evaluate long-term outcomes and cost-effectiveness.

**Supplementary Information:**

The online version contains supplementary material available at 10.1038/s41598-026-53658-2.

## Introduction

Preventive health screenings have emerged as an essential component of modern healthcare, offering opportunities to identify diseases early, often before symptoms appear^1–3^. With the increasing prevalence of noncommunicable diseases (NCDs), including cardiovascular diseases, diabetes, and metabolic disorders, the early detection through systematic screenings is fundamental to mitigating the long-term burden on patients and healthcare systems alike^4,5^. Proactively identifying health risks and conditions enables timely intervention, which is often associated with better prognoses, improved quality of life, and reduced healthcare costs^6–8^. Yet, despite these advantages, many conditions remain undiagnosed until later stages of life.

Preventive health programs provide important benefits, yet many conditions continue to go undiagnosed, reflecting limitations of routine evaluations, incomplete medical histories, and patients’ reluctance to seek care in the absence of symptoms^2,9–11^. Even when patients provide prior medical records or complete detailed anamneses, these sources may fail to capture emerging or undetected conditions, highlighting the limitations of relying solely on retrospective health data. Consequently, preventive screenings serve a dual purpose: not only do they confirm known health risks, but they also function as a crucial diagnostic tool to reveal previously “hidden” diseases^2,9,12^. This dual role formed the rationale for the German ‘Ü45-Check’ study, which offers comprehensive preventive examinations for adults in midlife.

Recent studies have highlighted the importance of regular health check-ups in addressing undiagnosed conditions, particularly those that are asymptomatic or slow to progress^13–16^. Elevated cholesterol levels, hypertension, and diabetes are among the conditions most frequently missed in routine care, yet readily detectable in preventive examinations^2,6,16,17^. Integrating clinical assessments into screening programs offers a robust framework for identifying these risks. However, evidence on the prevalence of previously undocumented suspected diagnoses in middle-aged adults is scarce, especially when using comprehensive screening protocols that combine medical histories, clinical examinations, and laboratory testing. Moreover, little is known about how clinical and behavioral factors contribute to such undetected conditions.

Although Germany already provides general health check-ups such as the Check-up 35, which provides a free examination every three years for everyone aged 35 and older, including a physical examination and reduced laboratory tests of blood, these are less comprehensive than programs that combine medical histories, clinical examinations, and laboratory testing^18^. Consequently, the nationwide implementation of more comprehensive preventive screenings continues to be debated, partly due to uncertainty about cost-effectiveness. In line with this, systematic reviews by Krogsbøll et al. have shown that general health checks do not significantly reduce morbidity or mortality^19-23^.

In the present secondary analysis of the ‘Ü45-Check’ study, we analyze data from 1,040 adults aged 45–59 years who underwent comprehensive preventive examinations, which is currently only offered as part of research projects. The Ü45 Check includes an extensive clinical examination including anthropometric measures like a Bioelectrical Impedance Analysis, a twelve-lead resting electrocardiogram, anamnesis, cardiovascular examinations, and blood samples and questionnaire survey^1^. In this secondary analysis of the study, our objectives were to quantify the prevalence of previously undocumented suspected diagnoses and to examine their associations with clinical and behavioral factors. Furthermore, we seek to evaluate the relationship between these diagnoses and key health indicators such as lipid profiles, blood pressure, and glycemic control, while considering the influence of patient-reported histories and prior medical records^24^.

In this study, “suspected diagnosis” refers to conditions identified for the first time during the preventive health screening. These were not previously documented in participants’ medical records or self-reported medical histories. As this is a cross-sectional survey, a definitive diagnosis was not established during the preventive health examination.

## Methods

### Study design and population

The present study is a secondary analysis of the prospective cross-sectional “Ü45-Check” study, which was originally designed to assess the need for prevention and rehabilitation based on preventive health examinations and a questionnaire survey. While the primary aim of the Ü45-Check was to evaluate overall health status in midlife, the rich dataset also allows for further insights beyond the initial objectives. In this analysis, we specifically examined the prevalence of previously undocumented suspected diagnoses and their associations with clinical and behavioral factors. Data collection included a combination of self-reported medical histories, prior to medical records provided by the participants, and objective findings from clinical evaluations. The screenings were offered to employees aged 45–59 years residing in Berlin and the federal state of Brandenburg, Germany. Participants were randomly selected by the German Pension Fund and invited for the “Ü45-Check” across four successive calendar years, from 2021 to 2024. After meeting the inclusion criteria of being insured by the Germany Pension Fund, residing in Berlin or Brandenburg and being between 45 and 59 years of age, and after not meeting any of the exclusion criteria (insufficient German or English language skills, already being in early retirement, or having recently undergone rehabilitation treatment), a total of 1,040 participants were included in the study^1^. Full details of the study design can be found in^1^. The present secondary analysis included all 1,040 participants from the original study sample and did not apply any additional exclusion criteria.

### Setting

The “Ü45-Check” was conducted as a preventive health examination within the outpatient clinic of the Department of Sports Medicine at Charité – Universitätsmedizin Berlin/Humboldt-Universität zu Berlin, Germany. The examination included clinical evaluations and a questionnaire survey targeting employees aged 45–59 years in Berlin and Brandenburg. The following exclusion criteria were applied: Individuals living outside of Berlin or Brandenburg; those lacking proficiency in German or English; individuals already in early retirement; and those who had recently undergone rehabilitation. In Germany, certain groups, such as self-employed individuals, civil servants (e.g., judges, soldiers), farmers, and doctors, are not insured by the German Pension Fund (GPF), these occupational groups were also excluded from the study. Although the study was regionally conducted, the recruitment via the German Pension Fund indicates that the sample is broadly comparable to employed populations in other German states.

Participants received immediate feedback regarding their examination and laboratory results, together with a personalized consultation in which a physician reviewed the findings, explained potential health risks, and provided recommendations for follow-up care^1^.

### Preventive health examination

The comprehensive preventive health examination lasted approximately 120 min. It incorporated various assessments, including anthropometric measures (height, weight, hip and waist circumference, bioelectrical impedance analysis (BIA)), handgrip strength, 12-lead resting electrocardiogram (ECG), systolic and diastolic blood pressure (SBP, DBP), anamnesis, and blood samples. The study design includes a detailed breakdown of all parameters, which can be found in Kalski et al. 2023^1^. The physical examination primarily evaluated the cardiovascular system, lungs, and abdomen. Blood samples were analyzed under fasting conditions.

A thorough medical history was taken during the subsequent consultation with the physician, focusing on study-specific details. Current health issues and medical needs were addressed, and further treatment and assessment recommendations were provided. In addition, the consultation covered counseling on regular exercise, health habits, and the importance of maintaining a healthy diet. As part of the anamnesis, participants were also asked whether they had undergone a blood test within the past 12 months. This variable was collected as an indicator of recent healthcare contact; under the assumption that more frequent medical testing may reduce the likelihood of undiagnosed conditions.

Suspected diagnoses were categorized into key health conditions such as elevated cholesterol, hypertension, diabetes, thyroid disorders, and other diseases (e.g., cardiac arrhythmias, asthma, thrombosis).

### Considered suspected diagnoses

#### Elevated cholesterol

Low-density lipoprotein (LDL) cholesterol is a key marker in assessing cardiovascular risk, with elevated levels (> 116 mg/dL) linked to atherosclerotic plaque formation^25^. As elevated LDL is typically asymptomatic, routine lipid profiling is valuable for early identification of individuals at risk of cardiovascular events^26,27^.

#### Hypertension

Hypertension is defined by a systolic blood pressure ≥ 140 mmHg or diastolic pressure ≥ 90 mmHg^28^ and is a major contributor to cardiovascular and cerebrovascular morbidity. Given its silent progression and high prevalence, blood pressure measurement is a low-cost and highly effective screening method for detecting latent cardiovascular risk^16,29^.

#### Diabetes mellitus

Diabetes mellitus involves chronic disturbances in glucose metabolism, and a high glycated hemoglobin A1c (HbA1c) level indicates prediabetic or diabetic states. However, diagnostic cut-offs vary across international recommendations. In this study, HbA1c levels above 6.0% were used as an indicator of potential dysglycaemia based on the National Institute for Health and Care Excellence (NICE) guidelines^30–32^. HbA1c testing provides a reliable long-term indicator of glycemic control, making it an efficient and non-fasting alternative for detecting undiagnosed metabolic disorders^33,34^.

#### Thyroid disease

Serum thyroid-stimulating hormone (TSH) levels (TSH 0.27 – 4.20 mU/l) serve as a sensitive indicator of thyroid function, with deviations from the reference range potentially indicating hypo- or hyperthyroidism. Due to the often vague and non-specific symptomatology of thyroid disorders, TSH screening enables early detection of dysfunctions that might otherwise remain undetected^35-37^.

#### Other diseases

In our study, this heterogeneous category includes clinically relevant conditions such as cardiac arrhythmias, heart diseases, thrombosis, and asthma, typically identified through physical examination, ECG, or medical history.

### Statistical analysis

**Fig. 1:**
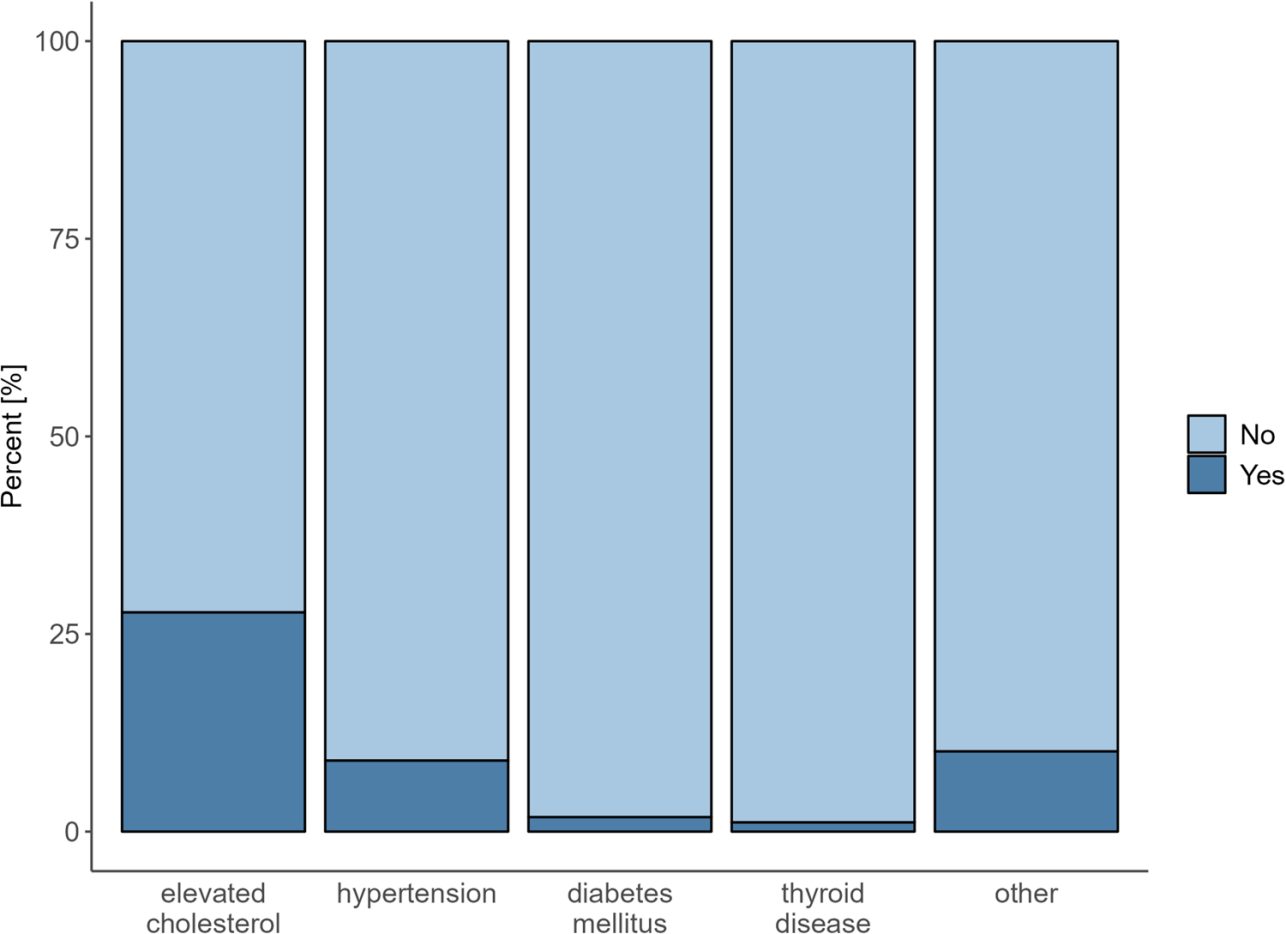
Barplot of newly suspected diagnoses identified during the preventive screening (n = 1,035; five participants had missing information on newly suspected diagnoses).

**Fig. 2:**
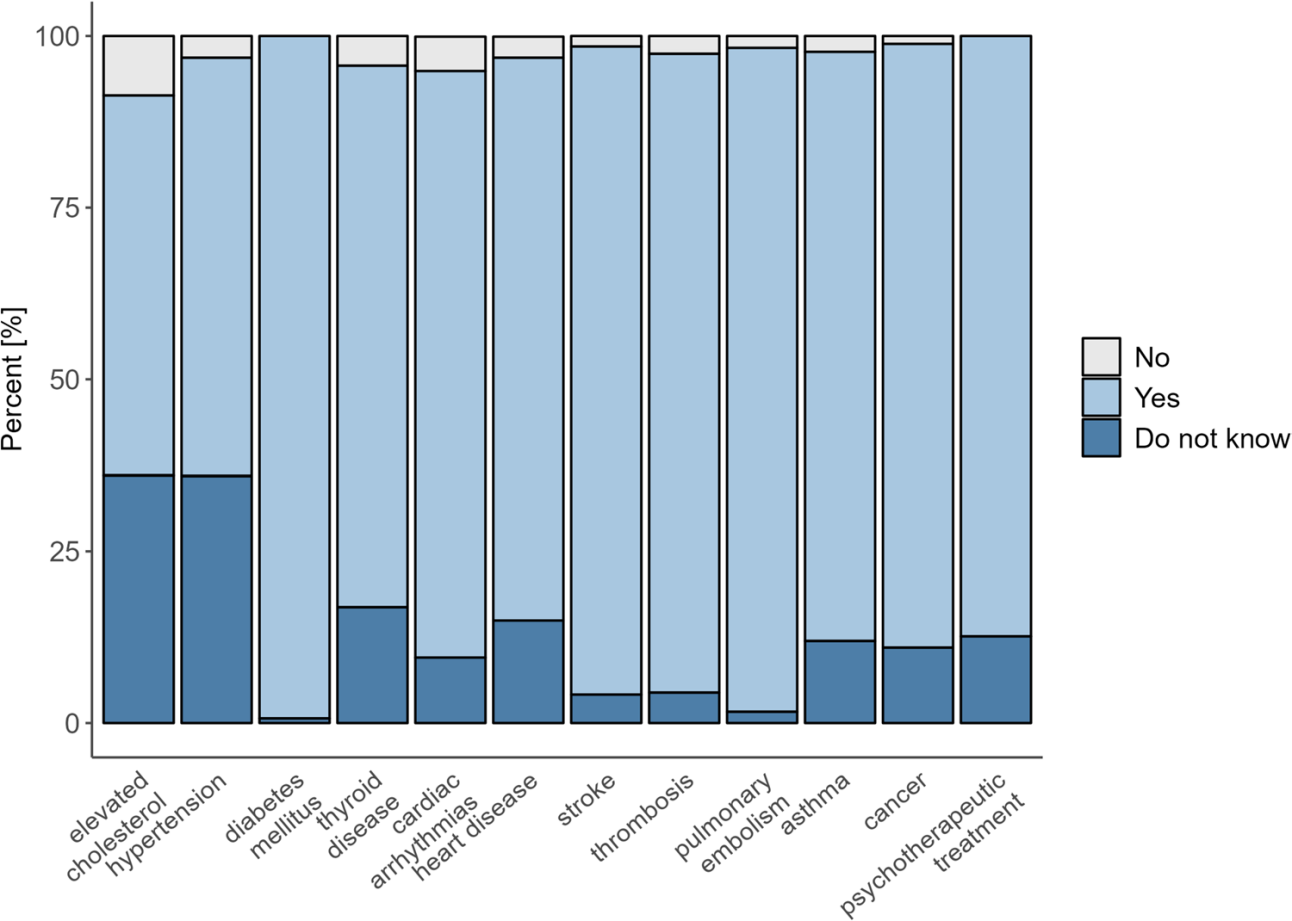
Barplot of self-reported or previously documented (pre-existing) diagnoses based on anamnesis or existing medical records (n = 1,038, for two participants; the data regarding the previously documented diagnoses is missing).

Initially, sociodemographic, anthropometric, and anamnestic characteristics were described using descriptive statistics, stratified by gender, presenting mean and standard deviation (sd) for continuous variables and absolute and relative frequencies for categorical variables (cf. Table 1). Descriptive tables stratified by the endpoints for any new suspected diagnosis, suspected hypertension and suspected elevated cholesterol are reported in the supplement (Tables S1, S2, S3). In a second step, both previously diagnosed and newly suspected diagnoses were reported by absolute and relative frequencies (cf. Table 2) and visualized with barplots (Figs. 1 and 2). To describe the association between the variables gender, Body-Mass-Index (BMI), current smoking status, alcohol consumption, and having at least one blood test in the last twelve months, as well as the amount of physical activity (categorized as “no sport”, “1–2 h/week”, and “more than 3 h/week”), on the occurrence of any new suspected diagnosis, a multivariable logistic regression model was estimated. The endpoint of any new suspected diagnosis includes all diagnosis regardless of their prevalence. Separate multivariable regression models were also estimated for the suspected diagnosis of hypertension and elevated cholesterol as outcomes. These two diagnoses had a sufficient number of cases to satisfy the rule of thumb of at least 10 events per variable. Participants with pre-existing diagnoses of hypertension or elevated cholesterol were excluded from the respective analyses. For the endpoint of any new suspected diagnosis, participants with a pre-existing diagnosis are not excluded, as they remain at risk of receiving another diagnosis of a different nature. The set of independent variables used in the general model with the combined endpoint of any suspected diagnosis was expanded for the disease-specific models: for hypertension, family history of vascular diseases and family history of heart disease were added; for elevated cholesterol, family history of dyslipidemia was included. Family history variables were not included in the composite endpoint model due to heterogeneity in availability and concerns regarding model stability. All regression results are presented as odds ratios (OR) and their corresponding 95%-confidence intervals (95%-CI) (cf. Table 3). Variable selection was guided by background knowledge, including prior research and clinical relevance, to ensure meaningful and interpretable model results. Model assumptions were checked using the variance inflation factor, Cook’s distance, and graphical representations of the predicted probabilities. No imputation methods were applied, given the low proportion of missing values (< 5%). A complete case analysis was performed, which resulted in reduced sample sizes in the regression models. All statistical analyses were conducted in R (version 4.4.2).

**Table 1:** Anthropometric, anamnestic, and socio-demographic characteristics.

Parameter N = 1,040	Total (mean (sd))	Male (mean (sd))	Female (mean (sd))
Gender (n (%))	1,040	631 (60.7%)	409 (39.3%)
Age [years]	52.93 (4.17)	52.93 (4.21)	52.93 (4.12)
Body-mass-index (BMI) [kg/m^2^]	26.89 (4.90)	27.45 (4.36)	26.02 (5.51)
Waist-to-hip-ratio (WHR)	0.90 (0.09)	0.95 (0.07)	0.82 (0.07)
Current smoking status (n = 1,038)			
No	797 (76.8%)	481 (76.0%)	316 (77.1%)
Yes	241 (23.2%)	149 (24.0%)	92 (22.9%)
Alcohol consumption (n = 1,038)			
No	163 (15.7%)	100 (15.8%)	63 (15.4%)
Yes	875 (84.3%)	530 (84.7%)	345 (83.6%)
Physical activity [hours/week]			
0 h	309 (29.7%)	194 (30.7%)	115 (28.1%)
1-2 h	355 (34.2%)	205 (32.5%)	150 (36.7%)
≥ 3 h	376 (36.2%)	232 (36.7%)	144 (35.2%)

**Table 2:** Suspected diagnoses.

Parameter	N = 1,035^1^ (%)
Suspected diagnosis	
No	614 (59%)
Yes	421 (41%)
Elevated cholesterol (LDL > 116 mg/dl)	
No	748 (72%)
Yes	287 (28%)
Hypertension (≥ 140/90 mmHg)	
No	942 (91%)
Yes	93 (9.0%)
Diabetes mellitus (HbA1c > 6.0%)	
No	1,016 (98%)
Yes	19 (1.8%)
Thyroid disease (TSH 0.27 – 4.20 mU/l)	
No	1,023 (99%)
Yes	12 (1.2%)
Other (e.g., Cardiac arrhythmia, Asthma, Thrombosis)	
No	930 (90%)
Yes	105 (10%)

HbA1c Glycated hemoglobin A1c; LDL Low-density lipoprotein; TSH Thyroid Stimulating Hormone. Suspected diagnoses refer to newly identified conditions during the preventive health examination that were not documented in prior medical records or self-reported histories^1^; five participants had missing information on newly suspected diagnoses.

**Table 3:** Multivariable logistic regression models for three clinical endpoints.

Variable	Any suspected diagnosis (n = 1,022)	Hypertension endpoint (n = 623)	Cholesterol endpoint (n = 565)
	Odds Ratio (OR) [95%-CI]		
Gender			
Female	reference	reference	reference
Male	1.42 [1.09; 1.85]	2.24 [1.31; 3.99]	1.30 [0.91; 1.86]
Body-mass-index (BMI)	1.01 [0.98; 1.04]	1.15 [1.08; 1.21]	1.04 [1.00; 1.08]
Current smoking status			
No	reference	reference	reference
Yes	1.11 [0.82; 1.50]	1.39 [0.79; 2.39]	1.15 [0.77; 1.71]
Alcohol behavior			
Never/abstinent	reference	reference	reference
Yes	1.09 [0.77; 1.56]	1.98 [0.94; 4.74]	1.12 [0.69; 1.85]
Physical activity [hours/week]			
0 h	reference	reference	reference
1-2 h	0.90 [0.65; 1.24]	1.0 [0.54; 1.83]	0.79 [0.51; 1.23]
3 h	1.13 [0.82; 1.56]	0.92 [0.50; 1.71]	1.02 [0.66; 1.58]
Last blood test ≤ 12 months ago	0.65 [0.50; 0.84]	0.77 [0.47; 1.26]	0.92 [0.66; 1.29]
Family history of vascular disease			
No	/	reference	/
Yes	/	0.88 [0.48; 1.56]	/
Do not know	/	1.77 [0.94; 3.25]	/
Family history of heart disease			
No	/	reference	/
Yes	/	1.33 [0.80; 2.24]	/
Do not know	/	1.90 [0.10; 13.0]	/
Family history of dyslipidemia			
No	/	/	reference
Yes	/	/	1.55 [1.01; 2.40]
Do not know	/	/	1.05 [0.71; 1.57]

Odds ratios (OR) and 95%-confidence intervals (CI) are shown for associations between selected predictors and three binary outcomes: composite endpoint, hypertension, and elevated cholesterol levels. Models are adjusted for all variables listed. Predictors not included in a given model are indicated by “/”, meaning the variable was not considered relevant for that specific outcome and was therefore excluded from the regression. Results are exploratory and interpreted descriptively.

### Ethics approval and consent to participate

Ethics approval was obtained from the “Ethics Committee of the Faculty of Culture, Social and Educational Sciences, Humboldt-Universität zu Berlin” on August 20, 2020 (reference number: HU-KSBF-EK_2020_0010). The work described was performed in accordance with the Declaration of Helsinki for experiments on humans. All subjects were informed by the study staff about the study procedure, subsequent data storage, and confidentiality, and pseudonymity of the data. Written informed consent was obtained from all subjects for the study center to use the data for research analyses and publication.

### Trial registration

The trial was registered at the German Clinical Trials Register (DRKS-ID: DRKS00030982). Retrospectively registered December 27, 2022, https://drks.de/search/de/trial/DRKS00030982.

## Results

Table 1 summarizes the anthropometric, anamnestic, and socio-demographic characteristics of the study population (N = 1,040), stratified by gender. The sample included 631 men (61%) and 409 women (39%), with a mean age of 52.93 years (sd = 4.17). Men had a higher average BMI of 27.45 kg/m^2^ (sd = 4.36) and Waist-to-Hip Ratio (WHR) of 0.95 (sd = 0.07), compared to women (BMI: 26.02 kg/m^2^ (sd = 5.51); WHR: 0.82 (sd = 0.07)).

Regarding smoking status, n = 241 (23.2%) of participants reported currently smoking, with a slightly higher prevalence among men n = 149 (24.0%) than women n = 92 (22.9%). The majority (n = 797, 76.8%) were non-smokers at the time of assessment. Alcohol consumption was reported by n = 875 (84.3%) of participants, with similar rates in men (n = 530, 84.7%) and women (n = 345, 83.6%). In contrast, 15.7% (n = 163) reported abstaining from alcohol, with nearly identical proportions among men (n = 100, 15.8%) and women (n = 63, 15.4%).

Physical activity levels were also similar across genders. Overall, n = 309, 29.7% of participants reported no weekly physical activity (men: n = 194, 30.7%, women: n = 115, 28.1%). About one third (n = 355, 34.2%) engaged in 1–2 h of activity per week, and n = 376, 36.2% reported being physically active for at least 3 h per week. It should be noted that the self-reported data likely reflect leisure-time physical activity.

Our cross-sectional survey of participants aged 45–59 years revealed numerous suspected diagnoses and risk factors for other diseases. As shown in Table 2 and Fig. 1, the study revealed important insights into the role of preventive health screenings in identifying previously undiagnosed conditions. In Fig. 1, the absolute and relative frequencies of suspected diagnoses first identified during the examination are visualized, corresponding to the data presented in Table 2. Five of the 1,040 patients had missing information on newly suspected diagnoses. Among the 1,035 participants, n = 421, 41% were found to have at least one new suspected diagnosis during their screening, highlighting the diagnostic value of such examinations. Elevated LDL cholesterol levels were the most common condition detected, affecting n = 287, 28% of the participants, followed by hypertension at n = 93, 9%. Other conditions, accounted for n = 105, 10% cases, most frequently cardiac arrhythmias and asthma, with fewer cases of thrombosis.

The data also indicated that conditions such as diabetes and thyroid disorders were less frequently diagnosed, with prevalence rates of n = 19, 1.8% and n = 12, 1.2%, respectively.

Figure 2 shows the distribution of known medical conditions reported by participants prior to the screening, based on anamnesis or existing medical records, which was available for 1,038 participants In contrast to the newly suspected diagnoses shown in Fig. 1, these values reflect conditions that were already known to participants before to the screening.

The most frequently reported diagnoses were elevated cholesterol (n = 374 (36%)) and hypertension (n = 373 (36%)). Other reported conditions included thyroid disease (n = 175 (17%)), heart disease (n = 155 (15%)), asthma (n = 124 (12%)), cancer (11%), cardiac arrhythmias (n = 99 (9.5%)), thrombosis (n = 46 (4.4%)), stroke (n = 43 (4.1%)), pulmonary embolism (n = 17 (1.6%)), and diabetes (n = 7 (0.7%)). In addition, n = 131 (13%) participants reported having undergone psychotherapeutic treatment at some point in the past.

Responses of “do not know” were most common for elevated cholesterol (8.7%), thyroid disease (4.3%), hypertension (3.2%), and heart disease (3.1%).

To complement these descriptive findings, multivariable logistic regression models were applied to examine associations between selected variables and three binary health outcomes: the composite endpoint any suspected diagnosis (n = 1,022), hypertension (n = 623), and elevated LDL cholesterol levels (n = 565). Table 3 presents odds ratios (OR) and 95%-CI for each variable. For the regression analyses of the hypertension and cholesterol endpoints, participants with a previously documented diagnosis of the respective condition were excluded. Accordingly, these models compare individuals with a newly suspected diagnosis identified during the examination to those without a prior diagnosis and without evidence of a new suspicion. In contrast, for the “any suspected diagnosis” outcome, no participants with pre-existing diagnoses were excluded, as individuals may still be at risk for additional previously undetected conditions. Therefore, this model reflects the association between covariates and the presence of any newly suspected condition in the screening examination, regardless of prior diagnostic history.

Gender was associated with any suspected diagnosis and hypertension. Compared with women, men had higher odds of presenting with suspected hypertension at the time of assessment (OR: 2.24; 95%-CI: [1.31; 3.99]) and for any suspected diagnosis (OR: 1.42; 95%-CI: [1.09; 1.85]) compared to women. No clear association was observed for elevated cholesterol (OR: 1.30; 95%-CI: [0.91; 1.86]). BMI was positively associated with the presence of suspected hypertension (OR: 1.15; 95%-CI: [1.08; 1.21]) and elevated LDL cholesterol levels (OR: 1.04; 95%-CI: [1.00; 1.08]), but showed no association with the composite outcome (OR: 1.01; 95%-CI: [0.98; 1.04]). Current smoking status and alcohol consumption were not clearly associated with any of the three endpoints. Alcohol use showed a tendency towards higher odds of hypertension (OR: 1.98; 95%-CI: [0.94; 4.74]), but the wide CI suggests that this result should be interpreted with caution. Physical activity levels were not meaningfully associated with any outcome. Odds ratios for both moderate (1–2 h/week) and higher (≥ 3 h/week) physical activity compared to no physical activity were close to 1 across all endpoints, with wide confidence intervals crossing 1. Participants who reported having a blood test within the last 12 months had lower odds for any suspected diagnosis (OR: 0.65; 95%-CI: [0.50; 0.84]). No associations were observed for hypertension or cholesterol in relation to this variable.

Family history variables were only included in the regression analyses, when deemed relevant for the endpoint (Table 3). Relevant positive associations among the family history variables were found for dyslipidemia with increased odds of elevated cholesterol levels (OR: 1.55; 95%-CI: [1.01; 2.40]). For family history of vascular disease, no association with hypertension levels was observed compared to participants without such a history (OR: 0.88; 95% CI: 0.48–1.56), whereas those who reported not knowing their family history showed a tendency toward higher odds compared to the reference group (OR: 1.77; 95% CI: 0.94–3.25).

These findings provide exploratory insights into associations between lifestyle and clinical characteristics and the presence of selected health conditions at the time of assessment. However, they do not permit causal interpretation and should be understood descriptively.

## Discussion

Preventive health screenings are a critical component of modern healthcare, offering an important opportunity to identify undiagnosed conditions before they progress to symptomatic or advanced stages^6,17^. In our cohort, the screenings revealed a substantial number of participants (n = 421, 41%) with previously unrecognized conditions, particularly in the domains of lipid metabolism, blood pressure regulation, and thyroid function. Although hypertension and elevated cholesterol were the most common known diagnoses, the screening additionally revealed a considerable number of previously undetected cases. Additionally, the high proportion of “do not know” responses for certain parameters, such as elevated cholesterol (8.7%), underscores gaps in prior diagnostic testing and/or health awareness. By systematically assessing individuals’ health through clinical examinations and the review of medical histories, these screenings provide the dual benefit of confirming known health risks and identifying previously undetected suspected conditions that may be relevant for long-term health outcomes, although not all findings will necessarily require immediate intervention^3,38^.

The findings of this study highlight the essential role of comprehensive preventive examinations in bridging the gap between patient-reported health information and objective clinical assessments^39^. The observed discrepancies between self-reported histories and screening findings are in line with previous research showing that self-reported prevalence often underestimates true disease burden. The ability to detect suspected conditions across domains such as lipid metabolism, blood pressure regulation, and metabolic or endocrine function highlights the diagnostic potential of these screenings^16^. Moreover, the results underscore the limitations of relying solely on prior medical records or self-reported histories, emphasizing the importance of integrating routine screenings into standard care. The lack of observed associations between lifestyle variables such as smoking, alcohol consumption, and physical activity with the defined endpoints should be interpreted in light of possible measurement limitations and limited statistical power. Physical activity was recorded as hours per week without a standardized definition, which may have led participants to include occupational or non-structured leisure activities. Similarly, alcohol and nicotine consumption were self-reported and may be underreported or misclassified, potentially influencing the regression model outcomes. In addition, these analyses were secondary and exploratory in nature and may have been limited by insufficient power. In particular, some categorical variables included small subgroup counts (see Tables S1–S3), resulting in wider confidence intervals and reduced precision of the estimates. Therefore, these findings should be considered hypothesis-generating rather than confirmatory. Associations identified in the regression models offer additional insights into characteristics associated with the presence of suspected conditions. Factors such as sex, BMI, and recent healthcare engagement (e.g., prior blood testing) showed patterns that may inform targeted screening approaches. The variable ‘recent blood test’ should, however, be interpreted as a proxy for healthcare utilization rather than a biological determinant. Its association suggests that individuals with less frequent healthcare contact may face a greater risk of remaining undiagnosed, which underlines the added value of low-threshold screening offers. While lifestyle variables such as smoking, alcohol consumption, and physical activity were not associated with the defined endpoints in this context, a family history of dyslipidemia showed a potential association with elevated LDL cholesterol. Other family history variables were selected based on clinical relevance and were not included across all models.

A population-wide screening program may also offer the advantage of reaching individuals who do not routinely seek medical care, thereby facilitating earlier detection of undiagnosed conditions in underserved groups. This is particularly relevant in light of our finding that 60% of participants were male, a group often reported to make less frequent use of routine preventive healthcare services^40–43^. Rather than directly explaining the observed detection rate, this demographic pattern may suggest that low-threshold screening programs are able to engage populations that are otherwise less likely to participate in routine care. The accessibility and low-threshold nature of population-based screening programs could therefore serve as a critical strategy to increase participation among men and other typically underrepresented groups in preventive healthcare. The fact that only a limited set of predictors showed consistent associations with suspected diagnoses further underscores the importance of offering the Ü45 screening broadly to all individuals in this age group, rather than restricting it to those with specific risk profiles.

However, screenings may also carry the risk of overdiagnosis, particularly when mildly abnormal or transient findings are classified as suspected conditions. This can lead to unnecessary follow-up, patient anxiety, or overtreatment, particularly in the absence of confirmatory diagnostics^44–46^. Therefore, the interpretation and communication of such findings require clinical caution and well-defined follow-up strategies.

In addition, the long-term cost-effectiveness of routine screenings remains a subject of debate. While early detection may improve quality of life and promote healthier behavior, systematic reviews by Krogsbøll et al.^19,20^ have shown limited effects on mortality or morbidity. Thus, screenings should be regarded as one component of a broader prevention strategy rather than as a universal solution.

To ensure the effectiveness of such initiatives, comprehensive public education and awareness campaigns are essential to address these challenges. These should address potential misconceptions, provide information on the benefits and limitations of screenings, and promote proactive health behavior to ensure broad participation and equitable access across diverse segments of the population^18,47,48^.

The results of this study have important implications for public health. They reinforce the need for policies that promote access to preventive health services, particularly among populations at risk of noncommunicable diseases^6^. By prioritizing early detection, healthcare systems can reduce the long-term burden of chronic diseases, improve patient outcomes, and optimize resource allocation^2,49^.

Future research should evaluate the long-term benefits of preventive health screenings, including their impact on morbidity, mortality, and healthcare expenditures. It should also explore strategies to minimize the risk of overdiagnosis while maximizing clinical relevance. Additionally, developing strategies to improve patient adherence to follow-up care and post-screening interventions will be crucial for maximizing the effectiveness of these programs.

### Strengths

This study has several strengths that enhance its relevance and impact on preventive healthcare. A key advantage is the large sample size of 1,040 participants, which provides a robust foundation for analyzing diagnostic patterns and strengthens the statistical reliability of the findings. This broad dataset provides meaningful insights into the frequency and distribution of suspected diagnoses within a diverse population.

The study also benefits from a comprehensive approach to data collection, combining self-reported health histories, prior medical records, and clinical findings^1,24^. This multidimensional methodology not only ensures a thorough evaluation of each participant’s health status but also highlights discrepancies between subjective reports and objective outcomes, offering a more nuanced understanding of the diagnostic process.

Moreover, the focus on real-world preventive screenings underscores the practical implications of the findings. By examining the outcomes of routine clinical evaluations, the study directly addresses the role of preventive measures in everyday healthcare, emphasizing their importance in early detection and intervention^2,50,51^. This real-world applicability further enhances the study’s relevance to clinical practice and policymaking.

Overall, the study provides valuable evidence that supports the diagnostic potential of preventive screenings, contributing to the broader understanding of their role in identifying hidden health conditions and improving long-term health outcomes.

### Limitations

In addition to its strengths, this study has several limitations that should be considered. First, the cross-sectional study design provides only a snapshot of diagnostic outcomes at a single point in time, preventing conclusions about causality or disease progression. Second, the study population comprised individuals who voluntarily participated in the preventive screenings, which may introduce selection bias and limit the generalizability of the findings to the broader population, particularly to those individuals less likely to engage in healthcare services. Third, information on current medication use was limited in our dataset and therefore could not be accounted for in the regression models. This limitation may introduce residual confounding, as pharmacological treatment may normalize clinical measurements and thereby affect the classification of newly diagnosed conditions. As a consequence, observed associations may be biased towards the null. Fourth, the use of self-reported health histories and prior medical records may be subject to recall bias or incomplete documentation, potentially affecting the accuracy of comparisons between known and newly identified conditions. In addition, the way certain parameters were assessed may have influenced the results. For example, physical activity was recorded as self-reported hours per week, which carries the risk that participants included occupational or general leisure activities not intended to be captured as structured exercise. A clearer, predefined definition of “physical activity” would help ensure consistent reporting in future studies. Similarly, alcohol and nicotine consumption were self-reported, and these behaviors may be underreported or misclassified. Such potential inaccuracies should be considered when interpreting the findings. Lastly, the screening focused on a predefined set of conditions (e.g., elevated cholesterol, hypertension), which, while clinically relevant, does not encompass the full spectrum of potentially detectable diseases. These limitations highlight the need for longitudinal studies and broader diagnostic scopes to fully assess the impact of preventive health examinations.

## Conclusion

This study underscores the diagnostic value of preventive health screenings in identifying previously unrecognized health conditions that may remain undetected in routine medical care. In our cohort, nearly half of all participants were found to have at least one suspected diagnosis, with elevated LDL cholesterol and hypertension being the most frequently detected conditions. The findings support the integration of systematic screenings into standard healthcare practices, particularly to bridge the gap between self-reported information and objective clinical indicators. By identifying such conditions early, screenings can inform timely interventions and contribute to better long-term health outcomes. However, to maximize their benefits, such programs must be carefully designed to avoid overdiagnosis, ensure appropriate follow-up, and reach populations typically underrepresented in preventive care. Future research should continue to evaluate the clinical impact, cost-effectiveness, and best practices for the implementation of these preventive strategies.

## Supplementary Information

Supplementary Information 1.

Supplementary Information 2.

Supplementary Information 3.

## Data Availability

The datasets used and/or analyzed during the current study available from the corresponding author on reasonable request.

## Abbreviations

BIA: Bioelectrical impedance analysis
BMI: Body-mass-index
CI: Confidence interval
DBP: Diastolic blood pressure
ECG: 12-Lead resting electrocardiogram
HbA1c: Glycated hemoglobin A1c
LDL: Low-density lipoprotein
NCD: Noncommunicable disease
OR: Odds ratio
sd: Standard deviation
SBP: Systolic blood pressure
TSH: Thyroid stimulating hormone
Ü45: People over 45 years old
WHR: Waist-to-hip ratio
