# Supplementary material for: Detection of previously undiagnosed conditions in midlife preventive health examinations
Source: Sci Rep. 2026 Jun 3;16:17132. doi: 10.1038/s41598-026-53658-2 (PMC13234109; doi:10.1038/s41598-026-53658-2)
Supplement: Supplementary file 1 — Supplementary Information 1. [file 41598_2026_53658_MOESM1_ESM.docx]

**Table S1** Descriptive values for all participants and variables entering the regression model with the endpoint any new suspected diagnosis.

| Parameter  N = 1,022 | No new suspected diagnosis  (n = 606)  (mean (sd)) | Any new suspected diagnosis  (n = 416)  (mean (sd)) |
| --- | --- | --- |
| Gender male (n (%)) | 344 (57%) | 273 (66%) |
| Body-Mass-Index (BMI) [kg/m²] | 26.79(5.11) | 27.05 (4.59) |
| Current smoking status |  |  |
| *no* | 470 (78%) | 313 (75%) |
| *yes* | 136 (22%) | 103 (25%) |
| Alcohol consumption |  |  |
| *no* | 99 (16%) | 61 (15%) |
| *yes* | 507 (84%) | 355 (85%) |
| Physical activity [hours/week] |  |  |
| *0h* | 175 (29%) | 123 (30%) |
| *1-2h* | 220 (36%) | 132 (32%) |
| *≥ 3h* | 211 (35%) | 161 (39%) |
| Last blood test ≤ 12 month ago |  |  |
| *no* | 241 (40%) | 211 (51%) |
| *yes* | 365 (60%) | 205 (49%) |
