# Supplementary material for: Detection of previously undiagnosed conditions in midlife preventive health examinations
Source: Sci Rep. 2026 Jun 3;16:17132. doi: 10.1038/s41598-026-53658-2 (PMC13234109; doi:10.1038/s41598-026-53658-2)
Supplement: Supplementary file 2 — Supplementary Information 2. [file 41598_2026_53658_MOESM2_ESM.docx]

**Table S2** Descriptive values for all participants and variables entering the regression model with the endpoint hypertension

| Parameter  N = 623 | No Hypertension  (n = 542)  (mean (sd)) | Hypertension    (n = 81)  (mean (sd)) |
| --- | --- | --- |
| Gender male (n (%)) | 289 (53%) | 60 (74%) |
| Body-Mass-Index (BMI) [kg/m²] | 25.27 (4.09) | 27.91 (4.46) |
| Current smoking status |  |  |
| *no* | 422 (78%) | 58 (72%) |
| *yes* | 120 (22%) | 23 (28%) |
| Alcohol consumption |  |  |
| *no* | 85 (16%) | 8 (10%) |
| *yes* | 457 (84%) | 73 (90%) |
| Physical activity [hours/week] |  |  |
| *0h* | 140 (26%) | 26 (32%) |
| *1-2h* | 180 (33%) | 28 (35%) |
| *≥ 3h* | 222 (41%) | 27 (33%) |
| Family history of vascular disease |  |  |
| *no* | 257 (47%) | 36 (44%) |
| *yes* | 208 (38%) | 24 (30%) |
| *do not know* | 77 (14%) | 21 (26%) |
| Family history of heart disease |  |  |
| *no* | 236 (44%) | 34 (42%) |
| *yes* | 301 (56%) | 46 (57%) |
| *do not know* | 5 (0.9%) | 1 (1.2%) |
| Last blood test ≤ 12 month ago |  |  |
| *no* | 260 (48%) | 45 (56%) |
| *yes* | 282 (52%) | 36 (44%) |
