# Supplementary material for: Detection of previously undiagnosed conditions in midlife preventive health examinations
Source: Sci Rep. 2026 Jun 3;16:17132. doi: 10.1038/s41598-026-53658-2 (PMC13234109; doi:10.1038/s41598-026-53658-2)
Supplement: Supplementary file 3 — Supplementary Information 3. [file 41598_2026_53658_MOESM3_ESM.docx]

**Table S3** Descriptive values for all participants and variables entering the regression model with the endpoint high cholesterol

| Parameter  N = 565 | Normal Cholesterol  (n = 326)  (mean (sd)) | High Cholesterol  (n = 239)  (mean (sd)) |
| --- | --- | --- |
| Gender male (n (%)) | 185 (57%) | 150 (63%) |
| Body-Mass-Index (BMI) [kg/m²] | 26.03 (4.73) | 26.86 (4.38) |
| Current smoking status |  |  |
| *no* | 251 (77%) | 177 (74%) |
| *yes* | 75 (23%) | 62 (26%) |
| Alcohol consumption |  |  |
| *no* | 47 (14%) | 33 (14%) |
| *yes* | 279 (86%) | 206 (86%) |
| Physical activity [hours/week] |  |  |
| *0h* | 82 (25%) | 68 (28%) |
| *1-2h* | 118 (36%) | 76 (32%) |
| *≥ 3h* | 126 (39%) | 95 (40%) |
| Family history of dyslipidemia |  |  |
| *no* | 133 (41%) | 87 (36%) |
| *yes* | 78 (24%) | 72 (30%) |
| *do not know* | 115 (35%) | 80 (33%) |
| Last blood test ≤ 12 month ago |  |  |
| *no* | 165 (51%) | 126 (53%) |
| *yes* | 161 (49%) | 113 (47%) |
